# Determinants of unmet need for family planning among married women of reproductive age in Burundi: a cross-sectional study

**DOI:** 10.1186/s40834-018-0062-0

**Published:** 2018-06-20

**Authors:** Athanase Nzokirishaka, Imose Itua

**Affiliations:** 1 Independent Consultant, Bujumbura, Burundi; 2 Department of Health Sciences, The University of Liverpool/Laureate Online Education, Liverpool, UK

## Abstract

**Background:**

High unmet need for family planning (32.4%) characterized Burundi in 2010. However, there has not been any study examining the relationship between unmet need and associated factors in Burundi. The present study aims at determining the demographic, socioeconomic and other factors underlying the unmet need for contraception among married women aged 15-49 in Burundi.

**Methods:**

This study used data from the 2010 Burundi Demographic and Health Survey. Total unmet need, unmet need for spacing and for limiting were used as outcomes and demographic, socioeconomic and other factors as independent variables. After a descriptive analysis of the study population (*n* = 5421), the association between the three outcomes and the independent variables were analysed using logistic regression. Odds ratios with their 95% confidence intervals were calculated with statistical significance at *p* < 0.05.

**Results:**

This study showed that the likelihood of total unmet need decreased with age after 35+, with an adjusted Odds Ratio = 0.586 and 95% CI = 0.423-0.811, compared to women aged 15-24. Women with 4-5 and 6+ living children had higher odds [aOR = 1.850 (1.322-2.590) and 2.390 (1.616-3.534) respectively]. Odds of unmet need were lower among women with primary [aOR = 0.741 (0.618-0.888)] and secondary education [aOR = 0.555 (0.399-0.771)]. Women whose husband desired more children than them [aOR = 1.824 (1.411-2.358)] and those ignoring the husband’s desired children [aOR = 2.700 (2.176-3.350)] had higher odds than those desiring the same number as the husband. Women who had experienced the death of 1+ sons had higher odds [aOR = 1.285 (1.038-1.591)]. Middle [aOR = 0.670 (0.530-0.846)] and rich [aOR = 0.664 (0.541-0.817)] compared to poor, women living in the North [aOR = 0.611 (0.412-0.904)] compared to those from Bujumbura, had lower odds. Rural women had higher odds [aOR = 1.373 (1.018-1.852)] and those who had visited a health facility [aOR = 0.765 (0.608-0.961)] or had access to TV [aOR = 0.562 (0.375-0.843)] had lower odds.

**Conclusion:**

Tackling the unmet need for FP in Burundi requires scaling-up male involvement, promoting spousal communication, client-centred services, greater use of media, women’s education, child survival, and pro-poor policies.

## Background

Unmet need for family planning designates “the number or percent of women currently married or in union who are fecund and who desire to either terminate or postpone childbearing, but who are not currently using a contraceptive method” [1].

Women with unmet need are composed of two groups: (a) Women with unmet for spacing: Those who desire to postpone their next birth by a specific length of time (for example, for at least 2 years from the date of a survey) and who do not currently use a contraceptive method.

(b) Women with unmet need for limiting: Those who desire no additional children and who do not currently use contraception.

Unmet need has been consistently used to monitor and evaluate sexual and reproductive health programmes in the framework of the Millennium Development Goals (MDGs) [2]. Compared to the contraceptive prevalence rate (CPR), an unmet need has the advantage of introducing a rights-based approach, as it captures at the same time the woman’s use of contraception and her fertility preferences.

Between 1990 and 2010, the CPR increased worldwide from 54.8 to 63.3% while unmet need decreased from 15.4 to 12.3%. Still, in 2010, the number of women with unmet need was estimated at 146 million out of which 122 million were from the developing countries, excluding China [3].

A high unmet need contributes to the perpetuation of high maternal mortality levels. Overall, while 342,203 women died of maternal causes in 2008, contraceptive use averted 272,040 (44%), and it is estimated that meeting the current unmet need could prevent an additional 29% of maternal deaths (104,000) per year worldwide [4].

Family Planning (FP) also impacts positively child survival and perinatal outcomes, through the lengthening of the inter-birth intervals. Children born within 2 years of an elder sibling have a 60% increased risk of infant death, compared with those born after 3 years or longer. Compared with inter-pregnancy intervals of 1.5 to 2 years, inter-pregnancy intervals shorter than 6 months induce an increase of 40% risks of preterm births and 60% risk of low birth weight [5].

Burundi is a small country with 27,384 km^2^. With a population of 8.4 million in 2010, it was the second most densely-populated country in Africa (310 inhabitants per square km). The urban population represented only 10%. The population grew by 2.4% a year, and the Total Fertility Rate (TFR) remained high at 6.4 children per woman. The Government of Burundi views FP primarily as a strategy to slow down the population growth, though it does not underestimate its health benefits.

FP programmes with modern methods started in Burundi in the early 1980’s and kept expanding over decades. Between 1990 and 2010, the CPR increased from 8.4 to 21.9% and unmet need increased slightly from 27.3 to 29.2% [3]. While unmet need decreased by 5.1% between 1990 and 2010 in developing countries, in Burundi, it increased slightly by 1.9% during the same period.

The research hypothesis of this study is that unmet need for contraception among women of childbearing age (15-49) is associated with demographic, socio-economic as well as knowledge, practice and exposure to media factors. The aim of the study is therefore to determine those factors underlying the unmet need for contraception in Burundi.

One such factor is the woman’s age*.* A study from Zambia [6] indicated that while unmet need increased below 34, it decreased with age, once a woman reached 34 years or more. The Zambian study also revealed that women who got married at 18+ had a lower likelihood of unmet need.

A study from India [7] highlighted that age at first marriage was significantly associated with unwanted pregnancy yet not associated with mistimed pregnancy while another from Burkina-Faso [8] discovered that being married more than once increased the likelihood of unmet need.

The number of living children or pregnancies (some authors use the terms “ever-born children” or parity) appears as a critical predictor of unmet need in many studies [8–10].

A study on the underuse of modern methods of contraception in 35 LMICs [11] revealed that women with the lowest educational attainment were 8.6 times less likely to use contraceptives compared with the highest levels.

One study from Cameroun [12] revealed that the husband’s approval of contraception and discussion about FP within the couple had a protective relationship with unmet need. A study from Rwanda [13] went on explaining that unmet need was higher among women who did not approve FP, those who believed their partner did not approve FP or who did not know his attitude and those who had never discussed FP with their partners or had done so, rarely.

Other factors found significantly associated with unmet need are low socioeconomic status, [14] the residence and region [15, 16], exposure to radio and knowledge of FP methods [17], exposure to information from health facilities [18] or counselling by a health worker [19].

In conclusion, this literature review highlighted that factors associated with unmet need vary across countries, hence the need to know which ones are at play and their relative importance in the context of Burundi. Such knowledge will inform the formulation and implementation of relevant policies, strategies, and interventions to tackle the unmet need in Burundi and expand contraception use.

## Method

This was a cross-sectional study based on the data collected by the 2010 Burundi Demographic and Health Survey (BDHS), during the period August 2010 – January 2011, under the authority of the National Statistical Office of Burundi (ISTEEBU), the National Public Health Institute (INSP) and ICF International. It was a population-based survey, aimed at updating the demographic, health and HIV-related indicators.

The 2010 BDHS used the 2008 Burundi Population and Housing Census (PHC) as the sampling frame. The sample was clustered by enumeration area (EA) and doubly stratified (by province and residence). More details are available in the BDHS final report.

All women of reproductive age found in the selected households as residents, or visitors who spent the previous night in the household, were eligible to be interviewed if they gave their informed consent. The present study was restricted to the sample of married women and those not married but living with a partner (*n* = 5421) because some of the questions in this study were related to the husband/partner’s characteristics.

The data collection used face to face interviews to administer the standard questionnaire developed by the MEASURE DHS Project adapted to the Burundi context. It was a structured and pre-tested questionnaire. The DHS programme uses three types of questionnaires: the household’s, the men’s and the women’s questionnaires. Data utilized in the present study come from the women questionnaire. The correspondent dataset file was downloaded from the DHS Measure project website and then opened directly into SPSS 21.0.

This study considered three outcomes: total unmet need, unmet need for spacing, and for limiting. The denominator for the calculation of unmet need is conventionally the total of currently married women aged 15-49 [20]. Divorced women and widows were excluded from the analysis. The numerator includes only women who were not using contraception at the time of the interview. Thus, the departing point for all calculations was a weighted sample of married women, who were first divided into those using a contraceptive method and those not using. The nonusers were first split into pregnant or amenorrhoeic women on one side, and those who were neither pregnant nor amenorrhoeic on the other. The pregnant or amenorrhoeic were then classified by whether the pregnancy or last birth was wanted at that time or unwanted. Women in the mistimed or unwanted category were considered having the unmet need for spacing and for limiting respectively. The other component of unmet need is composed of women who were neither pregnant nor amenorrhoeic. These women were further divided, into fecund and infecund. Fecund women who wanted to wait at least 2 years or who wanted no more children were regarded as having an unmet need for spacing and for limiting respectively.

Thus, the total unmet need was composed of unmet need for spacing plus the unmet need for limiting. The absolute number of women with unmet and met needs constitute the total demand for FP. The proportion of satisfied demand equals the percentage of the met need by the demand [1].

Independent variables were derived from the literature review and the 2010 BDHS questionnaire. They were mostly categorical and the few continuous, for example age, were categorized.

The wealth index was constructed by DHS program based on dwelling and household characteristics and assets. In this study, the five categories or quintiles of the wealth index (poorest, poorer, middle, richer, richest) were collapsed into three: poor regroups the bottom two quintiles and rich the top two quintiles. Middle remains unchanged.

A descriptive analysis examined the distribution of the sample of married women per demographic and socioeconomic characteristics as well as per their knowledge, practices, and exposure to media. Chi-squared tests with *p* < 0.05 were conducted to determine whether there was an association between an independent variable and an outcome. The univariate analysis allowed to assess the likelihood of total unmet need, unmet need for spacing and unmet need for limiting among women with certain characteristics using logistic regression to calculate unadjusted odds ratios with their 95% CI. A multivariate analysis, using logistic regression, examined the independent effect of each factor after controlling for confounders. Statistical significance was claimed if p was < 0.05 and CI did not span the unity. All *p*-values presented were based on two-tailed hypothesis.

The multivariate analysis was carried out on an unweighted sample of 3016 women who had an unmet need for spacing (*n* = 1701) or for limiting (*n* = 1315). Women who wanted another child in 2 years and infecund or menopausal were excluded from the analysis as they did not have an unmet need. An unweighted sample was used to respect the principle of matching a response with an individual. Adjusted Odds Ratios (aOR) with their 95% CI were estimated using two models:

- A multinomial logistic regression to analyze the unmet need for spacing (coded as 1) and the unmet need for limiting (coded as 2). The other category of women in the sample, coded as “met need” (coded 0), was considered as the reference category. A multinomial regression was appropriate as the outcome had more than two categories.
- A binary logistic regression to analyze the total demand for FP dichotomized into an unmet need (coded as 1) and met need (coded as 0) as a reference category.

## Results

### Differentials of unmet need

Results of descriptive analysis (Table 1) show that 64% of married women were aged below 35 years while 72.5% were aged 18 years and above when they got married or started cohabitating. Nine women (88.1%) out of ten had been married more than once and eight women out of ten (80.6%) had less than six living children.

**Table 1:** Demographic, socioeconomic and other characteristics of married women aged 15–49 years in Burundi, 2010

Characteristics (*n* = 5421)	Number	Percent
Woman’s age		
15–24	1307	24.1
25–34	2161	39.9
35+	1954	36.0
Age at first cohabitation		
< 18	1490	27.5
> 18	3931	72.5
Number of unions		
More than once	5523	88.1
Once	744	11.9
Number of living children		
0–1	1045	19.3
2–3	1935	35.7
4–5	1386	25.6
6+	1055	19.4
Woman’s education		
No education	2931	54.1
Primary	2145	39.6
Secondary +	344	6.3
Husband/partner’s education		
No education	2345	43.3
Primary	2571	47.4
Secondary+	448	8.3
Don’t know	57	1.0
Sex of head of household		
Male	4809	88.7
Female	611	11.3
Religion		
Catholic	3314	61.1
Protestant	1696	31.3
Muslim, others	412	7.6
Ideal number of children		
0–3	1552	28.6
4–5	2841	52.4
6+	1028	19.0
Husband’s desire for children		
Both same	2334	43.4
Husband wants more	715	13.3
Husband wants fewer	818	15.2
Don’t know	1510	28.1
Daughters dead		
0	4239	78.2
1+	1183	21.8
Sons dead		
0	4135	76.3
1+	1285	23.7
Wealth index		
Poor	2189	40.4
Middle	1129	20.8
Rich	2102	38.8
Respondent works for		
Family member	242	4.9
Someone else	499	10.1
Self–employed	4183	85.0
Respondent worked in last 12 months		
No	497	9.2
In the past year	493	9.1
Currently working	3980	73.4
Have a job but on leave last 7 days	450	8.3
Respondent currently working		
No	991	18.3
Yes	4430	81.7
Place of residence		
Urban	460	8.5
Rural	4961	91.5
Region		
Bujumbura	294	5.4
North	1625	30.0
Central–East	1397	25.8
West	993	18.3
South	1110	20.5
Visited by an FP worker		
No	5145	94.9
Yes	277	5.1
Visited a health facility		
No	1133	20.9
Yes	4288	79.1
Knowledge of any method		
Knows no modern method	43	0.8
Knows any modern method	5377	99.2
Heard FP in newspapers/magazine		
No	5309	98.0
Yes	107	2.0
Heard FP on radio		
No	2962	54.7
Yes	2455	45.3
Heard FP on TV		
No	5260	97.1
Yes	156	2.9

93.7% of respondents had primary education or no education at all, while 90.7% of women had husbands with primary or no education at all. Nine out of ten households (88.7%) were headed by males. 61.1% of participants were of the Catholic religion and 31.3% were Protestants. The ideal number of children for the majority of women (52.4%) was 4-5. While 43.4% of women desired the same number of children as their husbands, almost three women out of ten (28.1%) ignored their husband’s fertility preferences.

More than three-quarters of respondents (78.2%) had not experienced the death of a daughter and 76.3% had not experienced the death of a son. 61.2% of women belonged to the poor and middle categories of the wealth index, 85% were self-employed and 81.7% were working at the time of the interview. Participants were largely rural (91.5%) and concentrated in the North (30%) and the Central-East (25.8%).

A large majority (94.9%) had not been visited by an FP worker during the last 12 months preceding the interview. In contrast, 79.1% had visited a health facility during the same period. Practically, all women (99.2%) knew at least a modern contraception method while following proportions of women had not heard FP messages from newspapers/magazines (98%), radio (54.7%) and TV (97.1%).

### Prevalence of unmet need

Table 2 summarizes the distribution of the weighted sample of 5421 married women into six main groups: women with unmet need for spacing and for limiting, women with a met need for spacing and for limiting and women with no unmet need and infecund/menopausal women. Women with “met need” should not be confused with women with “no unmet need”. The former group refers to women using contraception, the latter to non-users. Among non-users, pregnant or amenorrhoeic women whose pregnancy or last child were wanted are considered as with “no unmet need”. So are not pregnant nor amenorrhoeic women wanting their next child within 2 years [16].

**Table 2:** Distribution of married women according to main categories of unmet need

	Percent	Number of women
Unmet need for spacing	22.6	(1225)
Unmet need for limiting	9.8	(531)
Met need for spacing	13.6	(735)
Met need for limiting	8.3	(451)
No unmet need	35.0	(1898)
Infecund, menopausal	10.7	(579)
Sub-total	100.0	(5419)
Missing	0.0	2
Total	100.0	(5421)

Unmet need among married women in Burundi stood at 32.4% (22.6% for spacing and 9.8% for limiting), against a met need of 21.9% (13.6% for spacing and 8.3% for limiting) in 2010. Thus, the total demand for FP (unmet need plus met need) was 54.3%. The proportion of satisfied demand was only 40.3% (37.6% for spacing and 45.9% for limiting).

### Bivariate analysis

The bivariate analysis provided a preliminary look at the characteristics of women with total unmet need, unmet need for spacing and for limiting, using a Pearson’s chi-squared test with a *p*-value < 0.05_._ Variables with *p* < 0.05 were selected to be included in the multivariate analysis (Tables 3, 4 and 5).

**Table 3:** Likelihood estimates of unmet need for spacing among married women aged 15–49 years in Burundi, 2010 (logistic regression analysis), *n* = 3016

Variables	OR (95% CI)	*p*-value	Adjusted OR (95% CI)	*p*-value
Woman’s age				
15–24	2.115 (1.690 –2.647)	0.000	3.048 (2.114–4.393)	0.000
25–34	2.043 (1.674–2.492)	0.000	2.436 (1.850–3.207)	0.000
35+ (reference)	1		1	
Number of unions				
Once	0.922 (0.684–1.244)		0.971 (0.690–1.366)	0.866
More than once (reference)	1		1	
Ideal number of children				
0–3	0.290 (0.225–0.374)	0.000	0.443 (0.330–0.595)	0.000
4–5	0.531 (0.423–0.667)	0.000	0.696 (0.534–0.907)	0.007
6+ (reference)	1		1	
Number of living children				
0–1	1.130 (0.845–1.511)	0.408	0.935 (0.606–1.443)	0.762
2–3	1.550 (1.212–1.982)	0.000	1.107 (0.786–1.560)	0.560
4–5	1.490 (1.149–1.932)	0.003	1.270 (0.931–1.732)	0.131
6+ (reference)	1		1	
Woman’s education				
No education	3.848 (2.938–5.038)	0.000	1.511 (1.006–2.270)	0.047
Primary	2.863 (2.187–3.748)	0.000	1.261 (0.862–1.846)	0.232
Secondary+ (reference)	1		1	
Religion				
Catholic	1.282 (0.971–1.691)	0.079	1.060 (0.764–1.469)	0.728
Protestant	1.643 (1.227–2.202)	0.001	1.188 (0.844–1.672)	0.323
Muslim, others (reference)	1		1	
Husband/partner’s education				
No education	5.187 (3.009–8.941)	0.000	0.792 (0.341–1.836)	0.586
Primary	4.251 (2.479–7.290)	0.000	0.696 (0.303–1.600)	0.394
Secondary+	1.791 (1.010–3.178)	0.046	0.681 (0.283–1.637)	0.391
Don’t know (reference)	1		1	
Husband’s desire for children				
Both same	0.297 (0.239–0.369)	0.000	0.385 (0.303–0.488)	0.000
Husband wants more	0.570 (0.423–0.769)	0.000	0.704 (0.509–0.975)	0.035
Husband wants fewer	0.342 (0.260–0.450)	0.000	0.450 (0.335–0.604)	0.000
Don’t know (reference)	1		1	
Daughters dead				
0	0.864 (0.698–1.070)	0.182	0.841 (0.653–1.084)	0.182
1+ (reference)	1		1	
Sons dead				
0	0.863 (0.700–1.063)	0.167	0.845 (0.662–1.080)	0.179
1+ (reference)	1		1	
Wealth index				
Poor	2.351 (1.963–2.817)	0.000	1.453 (1.148–1.839)	0.002
Middle	1.662 (1.333–2.071)	0.000	1.070 (0.820–1.396)	0.617
Rich (reference)	1		1	
Place of residence				
Urban	0.356 (0.291–0.434)	0.000	0.703 (0.501–0.987)	0.042
Rural (reference)	1		1	
Region				
South	3.393 (2.509–4.588)	0.000	1.139 (0.733–1.770)	0.563
North	1.613 (1.208–2.154)	0.001	0.585 (0.375–0.913)	0.018
Centre–East	2.685 (2.020–3.569)	0.000	0.859 (0.552–1.338)	0.502
West	4.226 (3.091–5.778)	0.000	1.321 (0.837–2.082)	0.231
Bujumbura (reference)	1		1	
Visited by a FP worker				
No	1.605 (1.100–2.343)	0.014	1.340 (0.879–2.042)	0.174
Yes (reference)	1		1	
Visited a health facility				
No	1.143 (0.923–1.415)	0.001	1.199 (0.928–1.548)	0.165
Yes (reference)	1		1	
Heard FP messages on radio				
No	1.737 (1.481–2.037)	0.000	1.228 (1.022–1.477)	0.029
Yes (reference)	1		1	
Heard FP messages on TV				
No	4.660 (3.207–6.772)		1.635 (1.017–2.629)	0.043
Yes (reference)	1		1

**Table 4:** Likelihood estimates of unmet need for limiting among married women aged 15–49 years in Burundi, 2010 (logistic regression analysis), *n* = 3016

Variables	OR (95% CI)	*p*-value	Adjusted OR (95% CI)	*p*-value
Woman’s age				
15–24	0.037 (0.020–0.069)	0.000	0.230 (0.097–0.547)	0.001
25–34	0.215 (0.169–0.273)	0.000	0.551 (0.398–0.762)	0.000
35+ (reference)	1			
Number of unions				
Once	0.542 (0.389–0.754)	0.000	0.830 (0.555–1.241)	0.364
More than once (reference)	1		1	
Ideal number of children				
0–3	0.953 (0.689–1.317)	0.769	1.793 (1.218–2.639)	0.003
4–5	0.726 (0.529–0.997)	0.048	1.283 (0.888–1.853)	0.185
6+ (reference)	1		1	
Number of living children				
0–1	0.012 (0.005–0.034)	0.000	0.017 (0.002–0.132)	0.000
2–3	0.085 (0.062–0.118)	0.000	0.172 (0.112–0.266)	0.000
4–5	0.361 (0.281–0.464)	0.000	0.521 (0.385–0.705)	0.000
6+ (reference)	1		1	
Woman’s education				
No education	6.757 (4.525–10.091)	0.000	1.696 (0.906–3.172)	0.098
Primary	2.612 (1.723–3.960)	0.000	1.035 (0.566–1.894)	0.910
Secondary+ (reference)	1		1	
Religion				
Catholic	1.202 (0.858–1.683)	0.284	0.996 (0.648–1.530)	0.985
Protestant	0.969 (0.671–1.401)	0.868	0.827 (0.520–1.314)	0.421
Muslim, others (reference)	1		1	
Husband/partner’s education				
No education	3.926 (2.173–7.094)	0.000	0.599 (0.172–2.085)	0.420
Primary	1.744 (0.964–3.157)	0.066	0.544 (0.157–1.889)	0.338
Secondary+	0.854 (0.442–1.648)	0.638	0.508 (0.138–1.869)	0.308
Don’t know (reference)	1		1	
Husband’s desire for children				
Both same	0.333 (0.254–0.437)	0.000	0.481 (0.352–0.659)	0.000
Husband wants more	0.780 (0.546–1.113)	0.170	0.845 (0.559–1.276)	0.423
Husband wants fewer	0.379 (0.268–0.537)	0.000	0.452 (0.305–0.671)	0.000
Don’t know (reference)	1		1	
Daughters dead				
0	0.355 (0.281–0.448)	0.000	0.729 (0.548–0.970)	0.030
1+ (reference)	1		1	
Sons dead				
0	0.321 (0.255–0.403)	0.000	0.748 (0.566–0.989)	0.042
1+	1		1	
Wealth index				
Poor	2.387 (1.906–2.990)	0.000	1.382 (1.013–1.884)	0.041
Middle	1.420 (1.063–1.896)	0.018	0.725 (0.502–1.048)	0.087
Rich (reference)	1		1	
Place of residence				
Urban	0.345 (0.264–0.452)	0.000	0.865 (0.550–1.361)	0.531
Rural (reference)	1			
Region				
South	4.146 (2.653–6.482)	0.000	1.127 (0.596–2.132)	0.713
North	2.536 (1.649–3.902)	0.000	0.776 (0.408–1.477)	0.440
Centre-East	4.293 (2.815–6.546)	0.000	1.192 (0.631–2.251)	0.589
West	5.825 (3.711–9.142)	0.000	1.459 (0.756–2.816)	0.260
Bujumbura (reference)	1		1	
Visited by a FP worker				
No	1.029 (0.670–1.580)	0.895	1.185 (0.704–1.995)	0.523
Yes (reference)	1		1	
Visited a health facility				
No	1.742 (1.359–2.233)	0.000	1.586 (1.166–2.156)	0.003
Yes (reference)	1		1	
Heard FP messages on radio				
No	1.340 (1.096–1.639)	0.004	0.993 (0.772–1.277)	0.957
Yes (reference)	1			
Heard FP messages on TV				
No	5.032 (2.937–8.622)	0.000	1.787 (0.883–3.616)	0.106
Yes (reference)	1		1

**Table 5:** Likelihood estimates of total unmet need among married women aged 15–49 years in Burundi, 2010 (logistic regression analysis), *n* = 3016

Variables	OR (95% CI)	*p*-value	Adjusted OR (95% CI)	*p*-value
Woman’s age				
15–24 (reference)	1		1	
25–34	1.119 (0.927–1.350)	0.243	0.800 (0.626–1.023)	0.074
35+	1.309 (1.076–1.593)	0.007	0.586 (0.423–0.811)	0.001
Age at first cohabitation				
< 18 (reference)	1			
≥ 18	0.866 (0.734–1.023)	0.090		
Number of unions				
Once (reference)	1			
More than once	1.313 (1.006–1.714)	0.045		
Number of living children				
0–1 (reference)	1		1	
2–3	1.497 (1.1901.833)	0.001	1.311 (0.986–1.743)	0.062
4–5	1.953 (1.540–2.477)	0.000	1.850 (1.322–2.590)	0.000
6+	2.705 (2.096–3.491)	0.000	2.390 (1.616–3.534)	0.000
Woman’ education				
No education (reference)	1		1	
Primary	0.609 (0.520–0.713)	0.000	0.741 (0.618–0.888)	0.001
Secondary+	0.218 (0.171–0.277)	0.000	0.555 (0.399–0.771)	0.000
Husband/partner’s education				
No education (reference)	1			
Primary	0.675 (0.574–0.795)	0.000		
Secondary+	0.296 (0.234–0.375)	0.000		
Don’t know	0.217 (0.140–0.335)	0.000		
Husband’s desire for children				
Both same (reference)	1		1	
Husband wants more	2.049 (1.606–2.614)	0.000	1.824 (1.411–2.358)	0.000
Husband wants fewer	1.147 (0.926–1.422)	0.210	1.107 (0.884–1.388)	0.375
Don’t know	3.256 (2.651–4.000)	0.000	2.700 (2.176–3.350)	0.000
Number of dead sons				
0 (reference)	1		1	
1+	1.670 (1.389–2.006)	0.000	1.285 (1.038–1.591)	0.021
Wealth index				
Poor (reference)	1		1	
Middle	0.670 (0.541–0.831)	0.000	0.670 (0.530–0.846)	0.001
Rich	0.423 (0.359–0.499)	0.000	0.664 (0.541–0.817)	0.000
Place of residence				
Urban (reference)	1		1	
Rural	0.352 (0.295–0.421)	0.000	1.373 (1.018–1.852)	0.038
Region				
Bujumbura (reference)	1		1	
North	1.840 (1.418–2.388)	0.000	0.611 (0.412–0.904)	0.014
Centre-East	3.080 (2.379–3.989)	0.000	0.930 (0.629–1.375)	0.715
West	4.619 (3.465–6.159)	0.000	1.406 (0.933–2.119)	0.104
South	3.578 (2.713–4.719)	0.000	1.161 (0.783–1.720)	0.457
Visited a health facility last 12 months				
No (reference)	1		1	
Yes	0.757 (0.625–0.917)		0.765 (0.608–0.961)	0.022
Heard FP in newspapers/magazine				
No (reference)	1			
Yes	0.301 (0.196–0.463)	0.000		
Heard FP on TV				
No (reference)	1			
Yes	0.210 (0.151–0.290)	0.000	0.562 (0.375–0.843)	0.005

### Multivariate logistic regression results

Table 3 shows that unmet need for spacing is significantly associated with women aged 15-24 [aOR = 3.048 (2.114-4.393] and 25-34 [aOR = 2.436 (1.850-3.207)] compared to women aged 35+. Women whose ideal number of children was between zero and three [aOR = 0.443 (0.330-0.595)] or four and five [aOR = 0.696 (0.534-0.907) were more protected against the unmet need for spacing than those whose ideal number of children was six and more.

Women with no education [aOR = 1.511 (1.006-2.270)] were more exposed to the unmet need for spacing than those with secondary and above education.

The likelihood of the unmet need for spacing was lower when both spouses desired the same number of children [aOR = 0.385 (0.303-0.488)], when the husband wanted more children than the wife [aOR = 0.704 (0.509-0.975)] or fewer children [aOR = 0.450 (0.335-0.604)], compared to when the woman ignored the number of children her husband desired.

In terms of economic factors, poor women [aOR = 1.453 (1.148-1.839)] had higher odds of unmet need for spacing than rich. Urban women [aOR = 0.703 (0.501-0.987)] compared to rural, women living in the Northern Region [aOR = 0.585 (0.375-0.913)] compared to those from Bujumbura had lower odds of unmet need for spacing.

Lastly, women who did not have access to FP messages through radio or TV were more exposed to unmet need for spacing with [aOR = 1.228 (1.022-1.477)] and [aOR = 1.635 (1.017-2.629)] respectively.

Results of the multivariate regression analysis (Table 4) show that unmet need for limiting was associated with old women aged 35+. Women aged 15-24 and 25-34 were less likely to experience it compared to those aged 35+ with respectively [aOR = 0.230 (0.097-0.547)] and [aOR = 0.551 (0.398-0.762)]. Women whose ideal number of children was zero to three [aOR = 1.793 (1.218-2.639)] were more likely to experience it, compared to those desiring six children and above. Similarly, the number of living children was found a key predictor of unmet need for limiting with respectively [aOR = 0.017 (0.002-0.132)), [aOR = 0.172 (0.112-0.266)] and [aOR = 0.521 (0.385-0.705)] for women with zero to one child, two to three and four to five children, compared to women with 6+ living children.

Women desiring the same number of children as the husband [aOR = 0.481 (0.352-0.659)] and those whose husband wanted fewer children [aOR = 0.452 (0.305-0.671)] were more protected against the unmet need for limiting than those who ignored their husband’s fertility preferences. This analysis also found a protective association between unmet need for limiting and having no experience of a girl death [aOR = 0.729 (0.548-0.970)] or a boy death [aOR = 0.748 (0.566-0.989)]. Poor women compared to rich [aOR = 1.382 (1.013-1.884)], women who had not visited a health facility during the last 12 months preceding the survey [aOR = 1.586 (1.166-2.156)] were found significantly associated with unmet need for limiting.

The number of unions, woman’s education, husband’s education, place of residence, region, exposure to radio or TV, which were univariately associated with unmet need for limiting lost significance in the multivariate analysis. Religion showed no association with unmet need for limiting neither in univariate nor in multivariate analysis.

Results of the multivariate logistic regression concerning total unmet need (Table 5) show that the likelihood of total unmet need decreased with the woman’s age, but the difference became significant after the woman was 35 years old, compared to women in the age group 15-24 [aOR = 0.586, (0.423-0.811)]. On the other hand, four to five and 6+ living children were found significantly associated with total unmet need with respectively [aOR = 1.850 (1.322-2.590)] and [aOR = 2.390 (1.616-3.534)]. The association between the number of unions and the husband/partner’s education with total unmet need found by the univariate model was lost in the multivariate model.

The multivariate model also revealed that total unmet need had a protective association with women educated at primary [aOR = 0.741 (0.618-0.888)] and secondary + [a OR = 0.555 (0.399-0.771)] levels. The husband/partner’s education which showed an association with total unmet need in the univariate analysis lost significance in the multivariate analysis. In contrast, the husband’s desire for children emerged as significantly associated with total unmet need both in univariate and multivariate models. The likelihood for total unmet need was higher among women whose husbands wanted more children (aOR = 1.824 (1.411-2.358)] or did not know the number of children desired by their husbands [aOR = 2.700 (2.176-3.350)], relative to women desiring the same number of children as the husband. The total unmet need was also found associated with women who had already experienced the death of a son or more, compared to those who had not [aOR = 1.285 (1.038-1.591)].

In the adjusted model, middle [aOR = 0.670 (0.530-0.846)] and rich [aOR = 0.664 (0.541-0.817)] categories of the wealth index had lower odds of total unmet need than the poor. Rural women compared to urban [aOR = 1.373 (1.018-1.852)] and women from the Northern Region compared to those from Bujumbura [aOR = 0.611 (0.412-0.904)] had higher and lower odds of total unmet need respectively. Lastly, the model detected that odds of the total unmet need for women who had visited a health facility during the last 12 months preceding the survey [aOR = 0.765 (0.608-0.961)] and those who had accessed TV messages [aOR = 0.562 (0.375-0.843)] were reduced.

## Discussion

This report highlighted that unmet need was higher for spacing (22.6%) than for limiting (9.8%), with a total of 32.4%. These estimates are consistent with those found in other reports: Using the revised definition of unmet need, another study [20] also estimated the total unmet need for Burundi at 32.4%. A third study [3], using a Bayesian hierarchical model combined with country-specific time trends to estimate the total unmet need of LMICs estimated the total unmet need for Burundi at 29.2% (95%CI: 20.0-39.7%). Lastly, the final 2010 BDHS report estimated the total unmet need at 31% (i.e. 21.5% for spacing and 9.5% for limiting). The difference with the present report is attributable to the fact that in the BDHS final report, non-users, pregnant or amenorrhoeic women and those whose pregnancy was due to a contraception failure were not included in the category of unmet need.

The multivariate analysis confirmed that other factors being constant, unmet need for spacing was significantly associated with young women between 15 and 34 and unmet need for limiting with older women aged 35+. The total unmet need was associated with women aged 35+ compared to women aged 15-24.

The husband’s desire for children has emerged as a dominant factor shaping unmet need for spacing, for limiting and total unmet need_._ Unmet need for spacing was less likely when both spouses wanted the same number of children, or the husband wanted more or fewer children compared to women ignoring their husband’s fertility intentions. The husband’s desire for children affected significantly unmet need for limiting when the two spouses wanted the same number of children or the husband wanted fewer children. It affected the total unmet need the same way. The ignorance of the husband’s desire for children can be an indication of lack of spousal communication on fertility and FP.

The sexual and reproductive health programme (SRHP) should involve males in all programming stages so that they can support their wife in using FP and inter-spousal dialogue on fertility and FP can be promoted [21].

The ideal number of children is another powerful factor underlying the unmet need for spacing and for limiting. The unmet need for spacing increases with the ideal number of children, meaning that the more children the women desire, the more they want to space them. On the other hand, unmet need for limiting was associated with women wanting a small family of zero to three children. These women seem to prefer to cease childbearing soon after their fertility goal is achieved. Further studies should confirm the likelihood of the unmet need for limiting among women desiring zero to three children as there is a possibility of a response bias. A maximum of three children per family being the norm in the official discourse, some women could have responded to the question about the ideal number of children by what is considered “politically correct”.

The multivariate analysis found out that the number of living children was significantly and positively associated with both unmet needs for limiting and total but not with the unmet need for spacing. The apparent association between the number of living children and unmet need for spacing, in the univariate analysis, was eliminated in the multivariate analysis, which means that the association in univariate analysis was due to confounders such as the ideal number of children. The association between unmet need and parity was found in many other studies. In Ethiopia, the number of living children was one of the factors affecting the unmet need for limiting [20] while in Uganda, it affected both the spacing, limiting and the total unmet need [22].

The multivariate analysis revealed the existence of a protective association between education and total unmet need and a positive association between women with no education and unmet need for spacing while no association was found between unmet need for limiting and woman’s education. These associations lost strength though, moving from the univariate to the multivariate analysis. The multivariate analysis showed that these findings were largely due to confounding. In Ethiopia [18], women with lower education were more likely to have both unmet needs for spacing and for limiting and in Nigeria [8] women with primary and secondary education were more likely to have an unmet need for spacing. These results point to the need to take the context into account when examining those associations.

The experience of the loss of a girl child was found significantly associated with unmet need for limiting and that of a son with both unmet needs for limiting and total unmet need. The death of a child, therefore, acted as a deterrent to the use of contraception for limiting. Women who lost a child tended to resume reproduction as soon as possible, to replace the loss. To be successful thus, SRH programmes need to be accompanied by efforts aimed at reducing child and infant mortality [22].

This study has shown that poor women compared to rich were at a higher risk of unmet need for spacing and for limiting and that women in the middle and rich categories were more protected against total unmet need compared to poor women. Rural residence increased the likelihood of the unmet need for spacing and total unmet need but played no significant role in limiting. Geographically, the multivariate analysis confirmed that the Northern region was better off in contraceptive use, compared to the Bujumbura region. This concentration of efforts in the North, which is also the most densely populated part of the country, could be a reflection that Government’s concern has been primarily demographic. While the region was found a strong predictor in the univariate analysis, it lost significance once confounders were controlled.

On the other hand, visiting a health facility affords the woman the opportunity to get counseling, information, and services and has shown association with unmet need for limiting and total unmet need. In this study, women exposed to radio or TV were more protected against the unmet need for spacing, but no relationship was detected with unmet need for limiting. However, the total unmet need was influenced by exposure to TV.

### Limitations

The fact that data for both the outcomes and predictors reflected the situation at one point in time prevents establishing causality relations between the dependent and independent variables, but rather a mere association. Causality supposes the anteriority of the exposure to the outcome, which is not the case in cross-sectional data.

The findings of this study have shown the importance of the man’s views on fertility and contraception in the Burundi male-dominated society. It is therefore important that further researches explore male views, a dimension which was missing in this research. On the other hand, although spousal communication about FP was deemed key in future interventions, the present study did not measure it directly for lack of relevant information in the questionnaire. It was just deducted from the importance of the group of respondents reporting to ignore husband’s desires. Male involvement and spousal communication constitute an area of interest for future studies on Burundi.

## Conclusion

This report examined the differentials, the prevalence, and factors associated with unmet need for FP among married women in Burundi in 2010. After controlling for other respondent’s characteristics, results indicate that women with unmet need for spacing were primarily younger, poor and rural, had no education, desired 6+ children, ignored their husband’s fertility preferences and were not exposed to radio or TV messages. Women from the Northern Region were better protected against the unmet need for spacing.

Unmet need for limiting was associated with poor, older women aged 35+, desiring zero to three children, having 6+ children and, ignoring their husband’s desire for children, having experienced the death of a daughter or a son and having not visited a health facility during the last 12 months before the interview.

Total unmet need was associated with poor, rural women, with no education, living with four to five or 6+ children, ignoring their husband’s fertility preferences or whose husband wants more children, who has already experienced the death of one or more sons, living in another region than the North, who has not visited a health facility during the 12 months preceding the survey and lacking access to TV messages.

Tackling the high unmet need for FP in Burundi requires multi-level interventions such as male involvement, promoting spousal communication, availing individual level specific services and information, increasing the use of media and designing and creating an enabling environment based on the promotion of women education, urbanization, and pro-poor policies.

## Abbreviations

BDHS: Burundi Demographic and Health Survey
LMICs: Low and middle-income countries
PHC: Population and Housing Census
SRHP: Sexual and reproductive health programme
TFR: Total Fertility Rate
